# CT-guided pulsed radiofrequency of sacral and pudendal nerves for herpes zoster–related neurogenic lower urinary tract dysfunction in male patients: a retrospective study

**DOI:** 10.3389/fneur.2026.1895335

**Published:** 2026-08-27

**Authors:** Haojia Li, Jingyu Liu, Hongsen Liao, Dianjun Wang, Yong Fei, Dejian Chen, Tao Wang, Ming Yao

**Affiliations:** 1 Department of Pain Medicine, Affiliated Hospital of Jiaxing University, Jiaxing, Zhejiang, China; 2 Department of Urology, Nanjing First Hospital, Nanjing Medical University, Nanjing, Jiangsu, China; 3 Department of Anesthesiology, Southwest Hospital, Army Medical University, Chongqing, China; 4 Department of Spinal Pain, Ningxiang Traditional Chinese Medicine Hospital, Ningxiang, Hunan Province, China

**Keywords:** herpes zoster, neurogenic lower urinary tract dysfunction, pudendal nerve, pulsed radiofrequency neuromodulation, sacral nerve

## Abstract

**Objectives:**

To evaluate the efficacy of CT-guided pulsed radiofrequency (PRF) neuromodulation targeting the sacral nerves (S2-S4) and the pudendal nerve in male patients with herpes zoster–related neurogenic lower urinary tract dysfunction (NLUTD), a condition lacking effective treatment and significantly impairing quality of life.

**Materials and methods:**

A retrospective analysis was conducted on 15 male patients with herpes zoster–related NLUTD who underwent CT-guided PRF treatment between 2024 and 2025. PRF was applied to the sacral nerve roots (S2-S4) and the pudendal nerve. Clinical outcomes were assessed using residual urine volume and Visual Analog Scale (VAS) scores for pain. Most patients received two to three treatment sessions. A 12-week follow-up was conducted, and quality of life was evaluated using the 36-Item Short Form Health Survey (SF-36).

**Results:**

PRF treatment resulted in significant and sustained improvements in both urinary function and pain. Residual urine volume decreased markedly from 174.7 ± 43.1 mL at baseline to 29.3 ± 42.2 mL at 12 weeks, while VAS scores declined from 5.8 ± 1.0 to 0.9 ± 1.1. Health-related quality of life also improved significantly, as reflected by reductions in KHQ (73.47 ± 7.17 to 27.27 ± 12.79) and IIQ-7 scores (17.40 ± 1.72 to 5.07 ± 3.01; *p* < 0.05). Clinical benefits were evident after the first session and further enhanced with repeated treatments, with most patients achieving near-complete symptom resolution at 12 weeks and no recurrence of urinary retention.

**Conclusion:**

CT-guided PRF neuromodulation of the sacral and pudendal nerves demonstrates significant therapeutic value for male NLUTD secondary to herpes zoster, providing durable improvements in pain relief, urinary function, and quality of life (SF-36) over a 12-week observation period. The dual neuromodulatory approach is safe and warrants further prospective validation.

## Introduction

Herpes zoster (HZ) is a painful dermatomal disorder caused by reactivation of latent varicella-zoster virus within the sensory ganglia. It typically presents with unilateral vesicular eruptions and acute neuropathic pain along the affected dermatome (1). A nationwide analysis in China reported an adjusted annual HZ incidence of 5.85 per 1,000 person-years. Among these patients, 15.84% developed postherpetic neuralgia(PHN), with the finding that older individuals had a higher risk (2). Beyond its characteristic cutaneous manifestations, HZ imposes a considerable economic and societal burden, attributable to its protracted clinical course and the associated decline in quality of life (3). Pathophysiologically, viral reactivation leads to inflammatory damage of the sensory and autonomic nerves, resulting in neuronal dysfunction. Among the various nerve segments that may be affected, sacral nerve involvement is rare but particularly challenging (4). When the sacral nerves are affected by viral infection, patients often develop vesicular eruptions and severe neuropathic pain in the perineal and perianal regions, which may be accompanied by urinary and fecal dysfunction (5). Despite the higher incidence of sacral herpes zoster in women, male patients experience urinary difficulties more frequently. This is especially true for older men with benign prostatic hyperplasia (6). This dysfunction stems from both pain-related voiding inhibition and virus-induced autonomic nerve injury, creating a dual pathology that is challenging to address with conventional treatments.

Neuromodulation techniques such as pulsed radiofrequency (PRF) and spinal cord stimulation (SCS) have shown efficacy in the management of neuropathic pain (7). However, these modalities primarily target somatic sensory pathways and are not intended to simultaneously modulate nociceptive transmission and the autonomic-motor circuits involved in bladder control. Urinary function is largely regulated by the autonomic nervous system particularly parasympathetic fibers originating from S2-S4 together with somatic motor innervation of the sphincters (8). Consequently, existing interventions fail to address the interplay between sensory pain modulation and autonomic regulation, highlighting the need for more precisely targeted neuromodulatory strategies capable of managing this complex, multifactorial dysfunction.

To bridge the existing therapeutic gap, we developed an innovative neuromodulatory strategy that integrates pulsed radiofrequency (PRF) applied to the sacral nerves (S2-S4) with concurrent PRF of the pudendal nerve. By targeting both autonomic and somatic pathways, this dual-site modulation aims to restore the coordinated regulation of bladder motility and urethral sphincter control while simultaneously alleviating neuropathic pain (9). Building on this rationale, we retrospectively evaluated male patients who underwent CT-guided PRF neuromodulation of the sacral and pudendal nerves at our center. This treatment has achieved notable improvements in both urinary function and pain relief, highlighting the clinical prospects of this combined approach as a minimally invasive and physiologically integrative intervention for neurogenic lower urinary tract dysfunction.

## Materials and methods

### Study design

This retrospective observational study included patients who visited our hospital between September 1, 2024, and September 1, 2025. The study protocol was approved by the institutional ethics committee and registered at chictr.org.cn (Registration No. 2025-LP-905).

### Participants

A total of 15 male patients diagnosed with neurogenic lower urinary tract dysfunction secondary to herpes zoster were enrolled based on the following inclusion and exclusion criteria. All participants provided written informed consent for the use and publication of their clinical data.

### Inclusion criteria

1. Male patients diagnosed with herpes zoster involving the sacral nerves (S2-S4), confirmed by characteristic dermatomal rash and clinical symptoms.
2. Presence of lower urinary tract dysfunction following herpes zoster infection, including symptoms such as urinary frequency, difficulty in voiding, urinary retention, or increased post-void residual volume.
3. Accompanying pain, numbness, or discomfort in the perineal, perianal, or genital region consistent with sacral nerve involvement.
4. No evidence of central nervous system lesions or other causes that could explain the urinary symptoms.
5. Patients with persistent urinary dysfunction despite standard medical treatment with antiviral agents and calcium-channel ligands (pregabalin or Crisugabalin)

### Exclusion criteria

Pre-existing neurogenic bladder or neurological disorders affecting bladder function (e.g., spinal cord injury, multiple sclerosis).

1. Severe systemic diseases or comorbidities that could interfere with treatment, assessment, or data interpretation.
2. Inability to cooperate with treatment or follow-up, or incomplete clinical data.
3. Inability to complete the 12 week postoperative follow-up.

### Procedure

Before undergoing PRF, all patients had received conservative medical therapy, including valaciclovir, methylcobalamin, pregabalin and tamsulosin, during outpatient management. Patients who failed to achieve satisfactory improvement were referred for hospitalization and further interventional treatment. During hospitalization, medications were continued, and flurbiprofen axetil was administered when necessary for severe pain. Opioids were avoided due to concerns regarding potential exacerbation of urinary retention. All procedures were performed under sterile conditions in a CT-guided intervention suite by a single experienced pain physician. Patients were placed prone with continuous vital monitoring. After local anesthesia, Sweet–Muller–Kerr (SMK) radiofrequency cannulas with a 10-mm active tip were advanced under intermittent CT guidance toward the S2–S4 sacral foramina and the pudendal nerve on the affected side. For pudendal nerve PRF, a posterior gluteal approach was used, with the target located at the level of the ischial spine, immediately dorsal to the attachment of the sacrospinous ligament. Needle trajectories were carefully adjusted under CT guidance to achieve accurate positioning while avoiding injury to the sciatic nerve and excessive advancement into the pelvic cavity. The needle trajectories were carefully adjusted to obtain accurate access to each target. Positioning was confirmed by impedance (250–550 *Ω*), negative aspiration, and sensory stimulation (100 Hz, ≤0.5 V) producing dermatomal paresthesia; motor testing ensured absence of lower-limb contraction. Pulsed radiofrequency was applied at 41 °C for 300 s per cycle (40-ms pulse width, 2 Hz), with two cycles per target. After reconfirming negative aspiration, a 10-mL injectate (lidocaine 0.5 mL, methylcobalamin 1 mL, betamethasone 4 mg, iohexol 1 mL, and normal saline to volume) was prepared and evenly divided for injection at each target site. A post-procedure CT scan verified epidural and pudendal-canal spread of the injectate. Cannulas were removed and puncture sites dressed. After a 30-min observation period, patients without complications were returned to the ward. Postoperatively, patients underwent a comprehensive assessment of pain intensity, spontaneous voiding ability, and post-void residual urine volume at the 1-week follow-up. Repeat PRF was performed when patients had persistent pain (VAS > 4), urinary retention after catheter removal, or a post-void residual urine volume >100 mL, based on the overall clinical evaluation.

### Urodynamics

All patients underwent post-void residual (PVR) volume assessment. Patients were instructed to empty the bladder completely, and urinalysis was performed to exclude urinary tract infection. PVR volume was measured by bladder ultrasonography immediately after voluntary voiding. The same evaluations were repeated at 1, 4, and 12 weeks after the first session of PRF treatment.

### Outcome measures

All outcome assessments were conducted at baseline (prior to the PRF procedure) and at 1, 4, and 12 weeks post-treatment. The evaluated domains included pain intensity, urinary symptoms, health-related quality of life, and psychological status.

### Pain intensity

Pain severity was assessed using the Visual Analog Scale (VAS), where patients rated their pain from 0 (no pain) to 10 (worst imaginable pain). The VAS score reflected herpes zoster-associated neuropathic pain. Treatment response was categorized based on the percentage reduction in VAS score from baseline as follows: excellent (≥75% reduction), good (50–75% reduction), fair (25–50% reduction), and poor (<25% reduction). The overall treatment response rate was defined as the proportion of patients achieving at least a fair response (≥25% reduction).

### Urinary symptoms and function

Urinary function was evaluated using both objective measures and patient-reported outcomes. Objective assessments included post-void residual (PVR) volume, measured via bladder ultrasonography. The subjective burden of urinary symptoms and their impact on daily life were measured using the King’s Health Questionnaire (KHQ). Specific focus was placed on items related to nocturia and incontinence. Additionally, the Incontinence Impact Questionnaire-Short Form (IIQ-7) was employed to quantify the impact of urinary incontinence on physical activity, social relationships, and emotional health; higher scores on the IIQ-7 indicate greater functional impairment.

### Health-related quality of life

Health-related quality of life was assessed using the 36-Item Short Form Health Survey (SF-36). Three domains-Physical Functioning, Bodily Pain, and Social Functioning were analyzed in detail, and the overall SF-36 score was calculated to reflect general health status. Assessments were performed preoperatively and at 1 and 4 and 12 weeks after treatment, with higher scores indicating better quality of life.

### Statistical analysis

Statistical analyses were performed using SPSS 26.0 (IBM Corp., Armonk, NY, USA). Continuous variables were assessed for normality using the Shapiro–Wilk test and are presented as mean ± standard deviation (SD) or median (interquartile range, IQR), as appropriate. Repeated-measures data with normal distribution were analyzed using repeated-measures analysis of variance (ANOVA), followed by Bonferroni-adjusted *post-hoc* comparisons, while non-normally distributed data were analyzed using the Friedman test with Bonferroni correction for pairwise comparisons. Categorical variables were analyzed using the chi-square test or Fisher’s exact test. A two-sided *p <* 0.05 was considered statistically significant.

## Results

### Patient characteristics

A total of 15 male patients with herpes zoster-related neurogenic lower urinary tract dysfunction were included in this retrospective study, and all patients completed the 12-week follow-up. The patients ages ranged from 47 to 79 years old, with a mean age of 68.1 ± 9.9 years. The median disease duration was 15 days (range, 6–42 days), indicating that most patients were in the acute to subacute stage of herpes zoster. Lesions were located on the left side in 9 patients (60.0%) and on the right side in 6 patients (40.0%). Voiding difficulty or urinary retention was observed in 13 patients (86.7%), while micturition-related pain was reported by 12 patients (80.0%). The median number of pulsed radiofrequency (PRF) sessions was 2 (range, 1–3). At baseline, the mean visual analog scale (VAS) score was 5.8 ± 1.0, and the mean residual urine volume was 228.67 ± 86.79 mL, indicating substantial urinary dysfunction accompanied by moderate pain. At 12 weeks after treatment, the overall treatment response rate reached 100%, with an average reduction in VAS score of 85.1% from baseline.

Overall, this cohort represents patients with herpes zoster-associated neurogenic bladder dysfunction characterized by prominent urinary symptoms and pain. The baseline characteristics of the patients are summarized in Table 1.

**Table 1:** Baseline characteristics of the patients (*n* = 15).

Characteristic	Value
Age (years), mean ± SD	68.1 ± 9.9
Age range (years)	47–79
Disease duration (days), median (range)	15 (6–42)
Lesion side (left), n (%)	9 (60.0%)
PRF procedures, median (range)	2 (1–3)
Voiding difficulty / urinary retention, n (%)	13 (86.7%)
Micturition-related pain, n (%)	12 (80.0%)
Preoperative residual urine (mL), mean ± SD	228.67 ± 86.79
Preoperative VAS score, mean ± SD	5.8 ± 1.0

**Table 2:** Changes in clinical outcomes before and after PRF treatment.

Parameter	Preoperative	1 week post-treatment	4 weeks post-treatment	12 weeks post-treatment
Residual urine volume (mL)	228.67 ± 86.79	110.00 ± 50.85*	57.67 ± 45.70*	29.33 ± 42.17*
VAS score	5.80 ± 1.01	3.40 ± 0.91*	1.73 ± 0.96*	0.93 ± 1.10*
KHQ score	73.47 ± 7.17	52.33 ± 15.12*	34.33 ± 13.40*	27.27 ± 12.79*
IIQ-7 score	17.40 ± 1.72	11.60 ± 2.80*	7.07 ± 2.66*	5.07 ± 3.01*

Values are presented as mean ± standard deviation (SD). *p < 0.05 compared with the preoperative value.

**Table 3:** Changes in SF-36 scores during the 12-week follow-up.

Variable	Preoperative	1 week	4 weeks	12 weeks
Physical Function	16.47 ± 1.96	21.73 ± 1.33*	23.40 ± 1.18*	24.13 ± 1.19*
Bodily Pain	4.61 ± 1.25	7.68 ± 1.32*	9.92 ± 1.26*	11.29 ± 0.93*
Social Function	5.00 ± 1.25	6.47 ± 1.25*	8.13 ± 0.64*	9.40 ± 1.12*
SF-36 Total Score	76.07 ± 3.09	95.88 ± 2.57*	104.45 ± 2.27*	109.83 ± 2.20*

Values are presented as mean ± standard deviation (SD). *p < 0.05 compared with the preoperative value.

### Treatment sessions and safety

**Figure 1:**
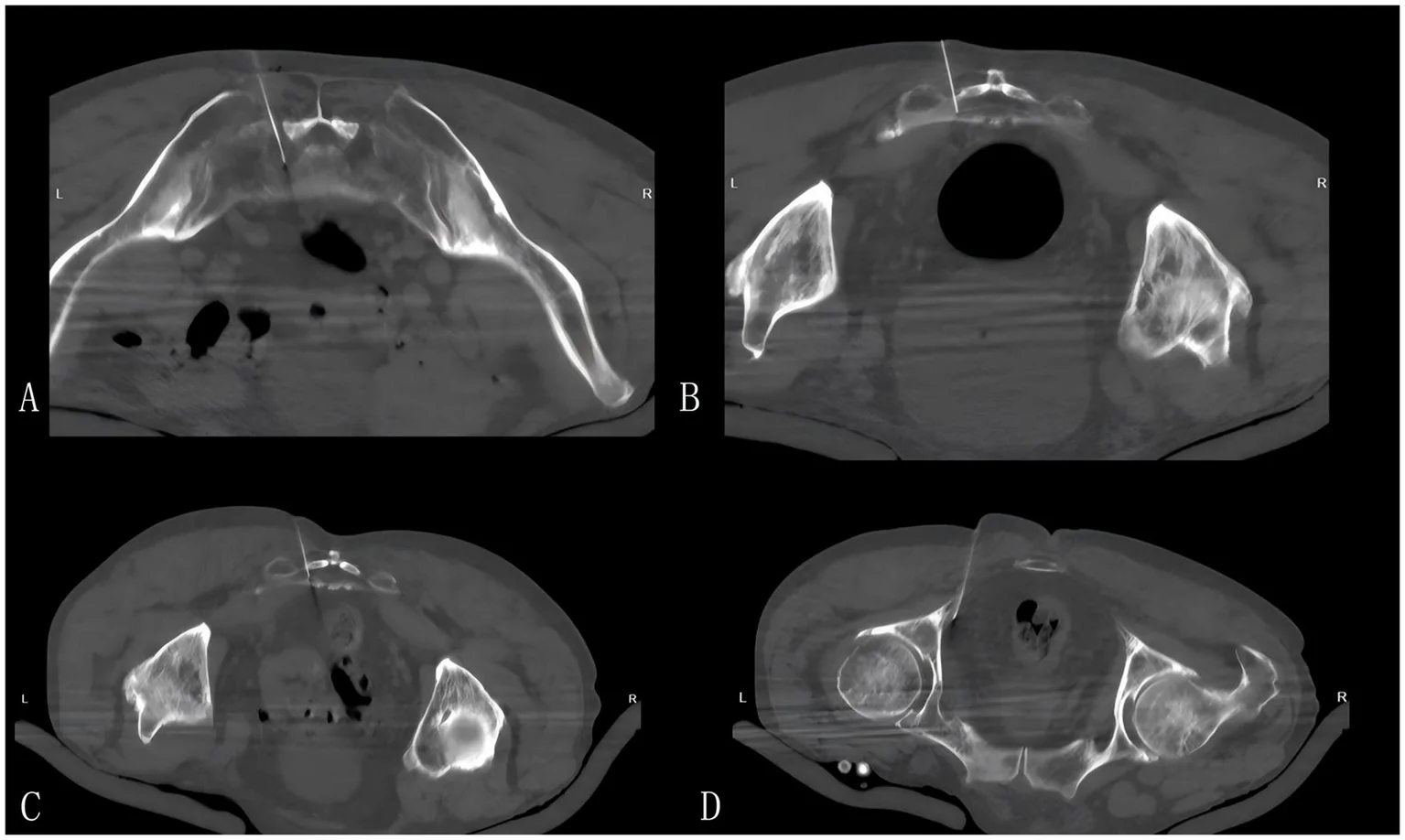
CT-guided needle placement for pulsed radiofrequency (PRF). **(A)** S2 sacral nerve; **(B)** S3 sacral nerve; **(C)** S4 sacral nerve; **(D)** pudendal nerve in the pudendal canal.

All patients were completed at least one session of combined pudendal nerve and sacral nerve pulsed radiofrequency (PRF) treatment. The puncture target sites are illustrated in Figure 1. All procedures were performed under CT guidance, ensuring precise needle placement and procedural safety.

Most patients required 2–3 PRF sessions to achieve optimal symptom control. Clinical improvement was typically observed after the first session and was further enhanced and stabilized with repeated weekly treatments.

No serious procedure-related complications or adverse events were reported during the follow-up period. Specifically, there were no cases of infection, persistent neurological deficit, symptom exacerbation, or worsening urinary retention. All procedures were well tolerated, supporting the safety, feasibility, and minimally invasive nature of CT-guided PRF therapy.

### Therapeutic effects on urinary function and pain

Results were demonstrated that all patients had a clear therapeutic response after pulsed radiofrequency (PRF) treatment. Both pain intensity and residual urine volume decreased markedly after the initial procedure, with further improvement observed following repeated weekly sessions. In terms of treatment frequency, 10 patients (66.7%) underwent two PRF sessions, 3 patients (20.0%) received three sessions, and 2 patients (13.3%) achieved symptom relief after a single treatment, indicating that 2–3 procedures were sufficient for most patients to achieve optimal clinical benefit.

Residual urine volume showed a progressive and sustained reduction over time, decreasing from 228.67 ± 86.79 mL preoperatively to 110.0 ± 50.8 mL at 1 week, 57.7 ± 45.7 mL at 4 weeks, and 29.3 ± 42.2 mL at 12 weeks. Compared with baseline, post-void residual urine volume was significantly reduced at the 12-week follow-up (mean difference, 199.3 mL; 95% CI, 148.8–249.9; Cohen’s d = 2.18; *p* < 0.001), indicating a very large treatment effect. Most patients achieved near-complete recovery of bladder emptying by week 12.

Similarly, VAS scores demonstrated a progressive decline throughout follow-up, decreasing from 5.8 ± 1.0 at baseline to 3.4 ± 0.9 at 1 week, 1.7 ± 1.0 at 4 weeks, and 0.9 ± 1.1 at 12 weeks. Relative to baseline, pain intensity was significantly reduced at 12 weeks (mean difference, 4.87; 95% CI, 4.28–5.45; Cohen’s d = 4.59; *p* < 0.001), suggesting that the treatment was effective. By the end of follow-up, most patients reported minimal or no residual pain, suggesting sustained analgesic efficacy (Table 2).

Overall, CT-guided pulsed radiofrequency treatment targeting the sacral nerves and pudendal nerve resulted in rapid and durable improvements in both urinary function and pain control, with repeated weekly sessions contributing to enhanced and sustained therapeutic efficacy.

### Health-related quality of life outcomes (KHQ)

Health-related quality of life, evaluated using the King’s Health Questionnaire (KHQ), demonstrated marked improvement following treatment. Baseline KHQ scores indicated substantial impairment, with a mean score of 73.47 ± 7.17. A significant reduction was observed as early as 1 week post-treatment (52.33 ± 15.12), representing rapid symptomatic relief. Continued improvement was noted at 4 weeks (34.33 ± 13.40) and was further sustained at 12 weeks (27.27 ± 12.79). The progressive decline in KHQ scores across all follow-up time points suggests sustained functional recovery and long-term improvement in urinary-related quality of life. Although variability increased at early follow-up, the overall downward trend remained consistent, indicating stable therapeutic benefit.

### Impact of urinary symptoms on daily life (IIQ-7)

**Figure 2:**
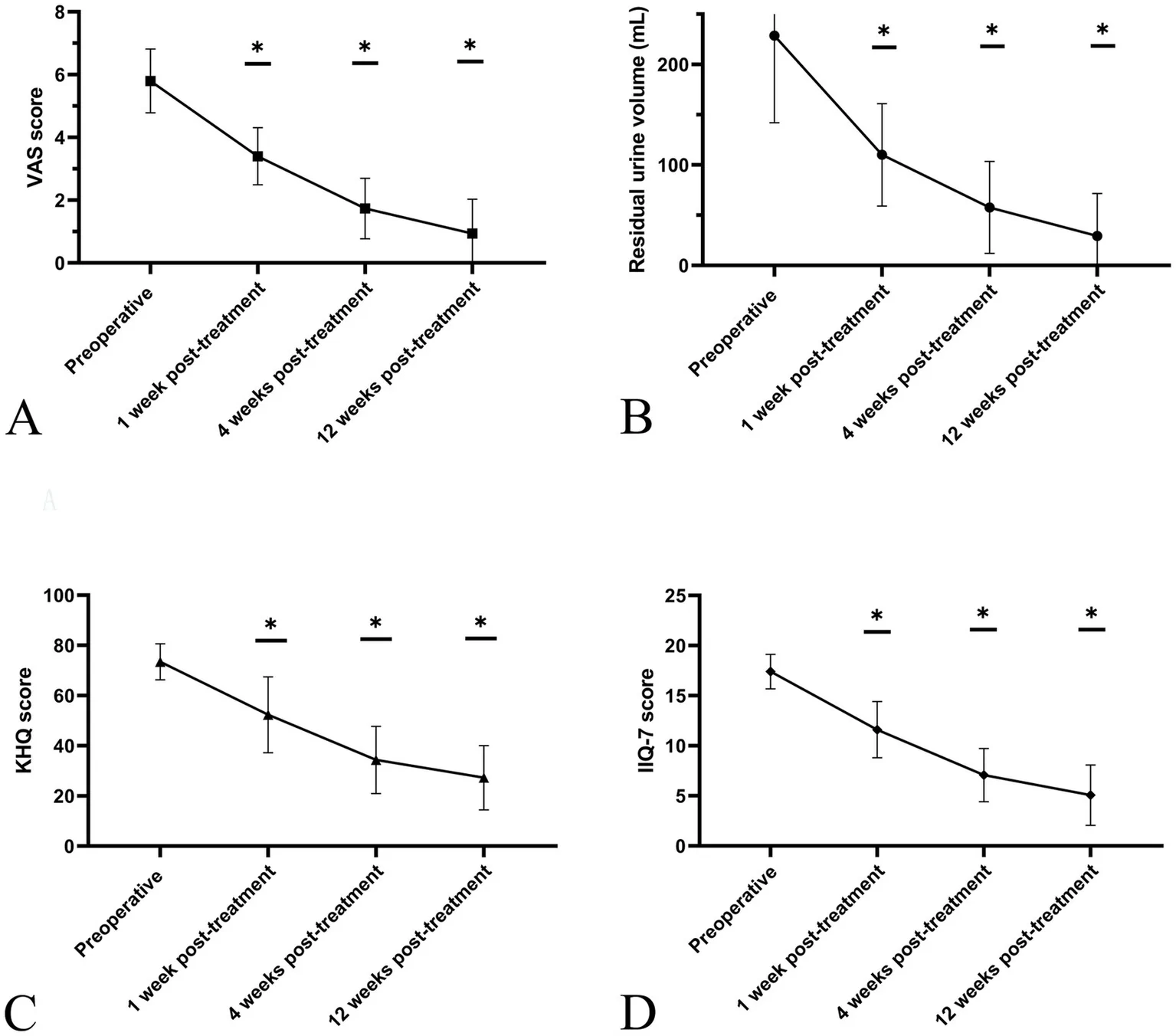
Changes in clinical outcomes following PRF treatment. **(A)** Visual analog scale (VAS) score; **(B)** Residual urine volume; **(C)** King’s Health Questionnaire (KHQ) score; **(D)** Incontinence Impact Questionnaire-7 (IIQ-7) score at baseline and during follow-up. *p* < 0.05 compared with the preoperative value.

The impact of urinary symptoms on daily activities, assessed using the IIQ-7, showed a parallel and progressive decline over time (Figure 2). The mean IIQ-7 score decreased from 17.40 ± 1.72 at baseline to 11.60 ± 2.80 at 1 week, further declining to 7.07 ± 2.66 at 4 weeks and 5.07 ± 3.01 at 12 weeks. This sustained reduction reflects significant alleviation of urinary-related restrictions in daily, social, and emotional functioning. The most substantial improvement occurred within the first month, followed by maintenance of therapeutic efficacy through week 12. The consistent improvement observed in both KHQ and IIQ-7 outcomes reinforces the positive impact of PRF treatment on overall health-related quality of life.

### Changes in SF-36 scores

Quality of life was evaluated using the SF-36 questionnaire over the 12-week follow-up period, with particular focus on Physical Functioning, Bodily Pain, and Social Functioning. As shown in Table 3, all assessed domains improved significantly over time (*p* < 0.001).

Physical functioning increased from 16.47 ± 1.96 at baseline to 21.73 ± 1.33, 23.40 ± 1.18, and 24.13 ± 1.19 at 1, 4, and 12 weeks, respectively (*p* < 0.05). Bodily pain scores improved from 4.61 ± 1.25 preoperatively to 7.68 ± 1.32, 9.92 ± 1.26, and 11.29 ± 0.93 during follow-up (*p* < 0.05). Social functioning similarly increased from 5.00 ± 1.25 to 6.47 ± 1.25, 8.13 ± 0.64, and 9.40 ± 1.12 (*p* < 0.05).

**Figure 3:**
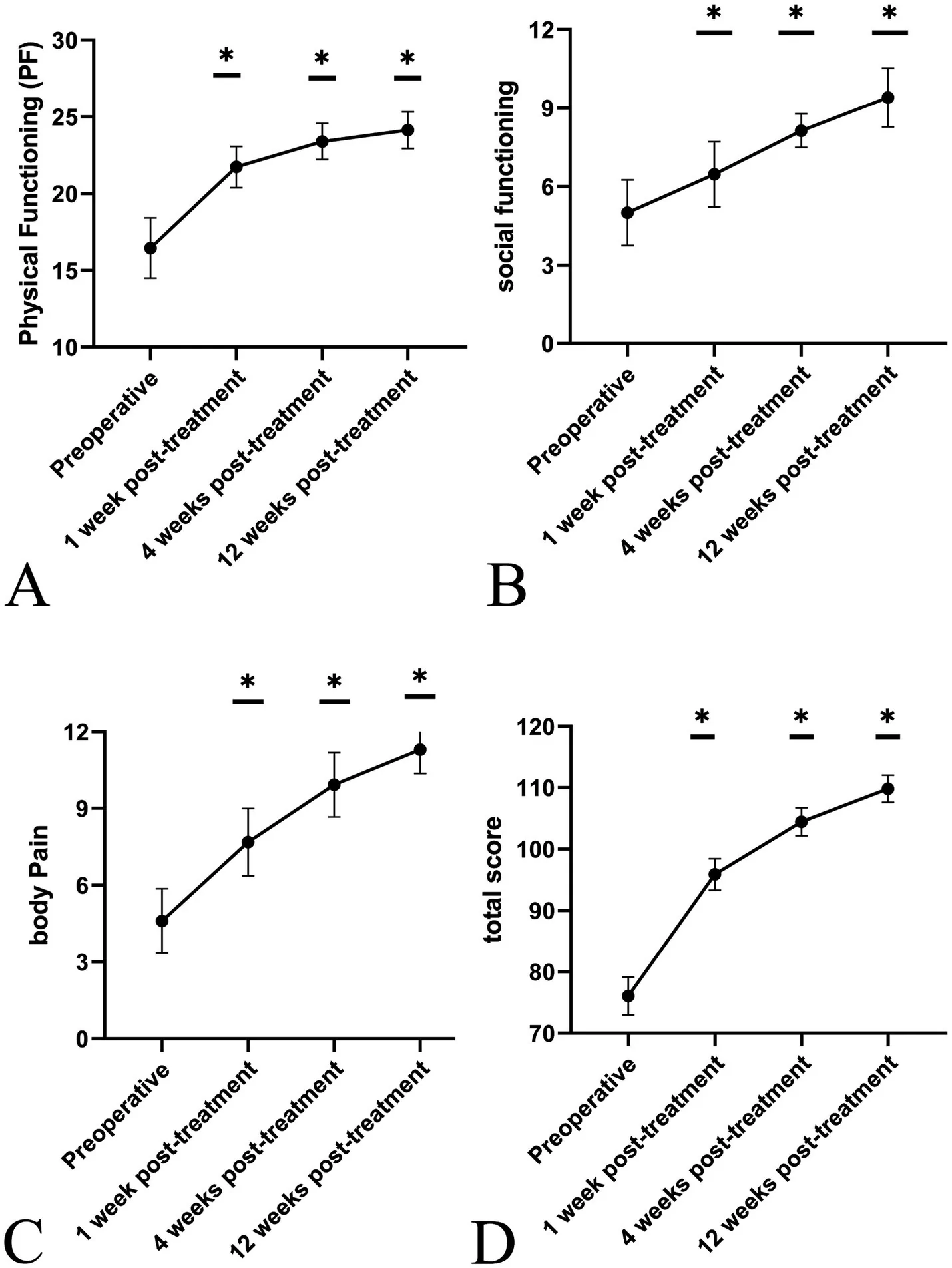
Changes in SF-36 scores during the 12-week follow-up. **(A)** Physical Functioning; **(B)** Social Functioning; **(C)** Bodily Pain; **(D)** SF-36 total score. Values are presented as mean ± SD. *p < 0.05 compared with preoperative values.

The SF-36 total score also showed a marked increase from 76.07 ± 3.09 at baseline to 95.88 ± 2.57, 104.45 ± 2.27, and 109.83 ± 2.20 at 1, 4, and 12 weeks, respectively (all *p* < 0.05; Figure 3).

## Discussion

Herpes zoster (HZ) is a neurotropic viral disease caused by the reactivation of latent varicella-zoster virus in dorsal root ganglia (10). Although postherpetic neuralgia is the most widely recognized sequela, involvement of the sacral nerve roots can lead to a broad spectrum of urogenital and anorectal dysfunctions (11). In particular, male neurogenic lower urinary tract dysfunction (NLUTD) following sacral herpes zoster remains underrecognized and undertreated, despite its substantial impact on quality of life (12). The present retrospective study demonstrates that CT-guided pulsed radiofrequency (PRF) of sacral and pudendal nerves is a safe and effective intervention for improving urinary function, relieving pain, and enhancing quality of life in male patients with HZ-related NLUTD.

Herpes zoster-induced neural injury results from a combination of viral replication, immune-mediated inflammation, and secondary demyelination or axonal degeneration (13). When the sacral nerve roots (S2-S4) are involved, patients may present with vesicular eruptions and neuropathic pain in the perineal or genital region, often accompanied by urinary retention, dysuria, urgency, or incomplete bladder emptying. These symptoms are particularly prevalent in elderly male patients, perhaps due to age-related neural vulnerability and pre-existing subclinical bladder outlet dysfunction (14). In the present cohort, more than half of the patients had documented benign prostatic hyperplasia (BPH) before the onset of herpes zoster. Pre-existing BPH may have predisposed these patients to more severe urinary dysfunction or exacerbated urinary retention following sacral herpes zoster.

The mechanisms underlying HZ-associated voiding dysfunction are multifactorial (15). Normal voiding depends on a highly integrated neural network involving parasympathetic, sympathetic, and somatic pathways (16). The sacral parasympathetic system mediates detrusor contraction, whereas the pudendal nerve governs external urethral sphincter control and contributes to afferent sensory input from the perineum. Importantly, the pudendal nerve also contains sympathetic components that may modulate urethral resistance and pelvic floor tone (17). Disruption at any level of this network may lead to clinically significant voiding dysfunction. This anatomical and functional complexity underscores the need for therapeutic strategies capable of modulating multiple neural components simultaneously rather than targeting a single pathway (18).

Pulsed radiofrequency (PRF) represents a potentially neuromodulatory intervention in this context (19). Unlike continuous radiofrequency thermocoagulation, PRF delivers intermittent high-frequency electrical currents while maintaining tissue temperatures below 42 °C, thereby avoiding irreversible neural injury (20). This non-destructive property allows PRF to modulate neural activity without compromising structural integrity, making it particularly suitable for functional disorders involving mixed sensory, motor, and autonomic dysfunction (21). While PRF has been widely used in the management of neuropathic pain, including postherpetic neuralgia, its application in NLUTD remains relatively unexplored.

In the present study, CT-guided pulsed radiofrequency (PRF) targeting the sacral and pudendal nerves was associated with consistent and clinically meaningful improvements across multiple domains. Residual urine volume decreased significantly after a single treatment session, indicating enhanced bladder emptying efficiency, likely attributable to improved detrusor contractility and/or reduced outlet resistance. Neuropathic pain was markedly alleviated, which may have reduced fear-avoidance behavior and facilitated more effective voiding. In addition, partial restoration of urinary sensation may reflect recovery of afferent signaling.

During intraoperative motor stimulation, patients frequently reported sensations of bladder contraction or urinary urgency, providing indirect functional which may indirectly suggest that the targeted neural pathways are involved in bladder regulation and are responsive to neuromodulation. Although pharmacological therapies, including corticosteroids and lidocaine, play a role in pain control, our previous clinical observations indicate that medication alone provides limited benefit in improving urinary retention or voiding dysfunction. Nevertheless, given the retrospective design and the absence of a control group, spontaneous recovery of herpes zoster-associated neurogenic lower urinary tract dysfunction cannot be completely excluded.

While a single PRF session produced substantial improvement, repeated treatments appeared to further enhance and stabilize therapeutic outcomes. Most patients required two to three sessions to achieve near-complete symptom resolution, with sustained benefits observed at 3 months and no recurrence of urinary retention. Importantly, no serious adverse events were observed, supporting the safety and tolerability of this minimally invasive approach. These findings suggest that PRF may exert both immediate and cumulative neuromodulatory effects, contributing not only to analgesia but also to functional recovery of bladder control (20).

The mechanisms underlying the potential therapeutic effects of PRF in HZ-related NLUTD remain speculative. PRF may modulate abnormal afferent sensory signaling by reducing ectopic neural discharges and normalizing sensory transmission, thereby facilitating recovery of micturition reflexes. In addition, the anti-inflammatory and neuroprotective properties of PRF may contribute to neural recovery after herpes zoster-associated nerve injury. However, these mechanisms remain hypothetical because no urodynamic, neurophysiological, or mechanistic assessments were performed in the present study. Further experimental and prospective clinical studies are required to validate these proposed mechanisms.

An important observation in this study is the apparent benefit of early intervention. Most patients were treated within 1 month of HZ onset, a period during which neural injury may still be partially reversible. Early neuromodulation may prevent irreversible axonal degeneration, maladaptive plasticity, and central sensitization, thereby improving long-term outcomes. Clinically, this highlights the importance of early recognition of urinary dysfunction in patients with sacral HZ and timely referral for interventional management. Beyond improvements in objective urinary parameters, patients experienced meaningful gains in quality of life, including reduced nocturia, improved continence, better sleep, and decreased psychological distress, underscoring the broader impact of effective treatment.

### Limitations

This study has several limitations. First, the retrospective design and relatively small sample size limit the generalizability of our findings. Second, the absence of a control group precludes definitive conclusions regarding causality. Third, urodynamic studies were not systematically performed, which would have provided more objective insight into detrusor and sphincter function.

Future prospective, randomized controlled trials with larger cohorts and longer follow-up periods are warranted. Incorporating urodynamic assessments, neurophysiological testing, and imaging biomarkers may further elucidate the mechanisms of PRF in NLUTD. Additionally, comparative studies evaluating PRF against sacral nerve stimulation or pharmacological interventions would help define its optimal clinical role.

## Conclusion

CT-guided PRF targeting the sacral and pudendal nerves appears to be a safe and effective minimally invasive treatment for HZ-related NLUTD. By simultaneously modulating pain, sensory input, and motor coordination, PRF addresses key pathophysiological components of this condition and offers a promising neuromodulatory strategy, particularly when applied early in the disease course.

## Data Availability

The original contributions presented in the study are included in the article/supplementary material, further inquiries can be directed to the corresponding author.
